# Damage to the myogenic differentiation of C2C12 cells by heat stress is associated with up-regulation of several selenoproteins

**DOI:** 10.1038/s41598-018-29012-6

**Published:** 2018-07-13

**Authors:** Jiayong Tang, Aihua He, Hui Yan, Gang Jia, Guangmang Liu, Xiaoling Chen, Jingyi Cai, Gang Tian, Haiying Shang, Hua Zhao

**Affiliations:** 1 Animal Nutrition Institute, Sichuan Agricultural University, Chengdu, Sichuan 611130 China; 2 Trace Element Research Center, Sichuan Agricultural University, Chengdu, Sichuan 611130 China; 3 Department of Animal Sciences, Purdue University, West Lafayette, IN 47907 USA

## Abstract

This study was conducted to profile the selenoprotein encoding genes or proteins in mouse C2C12 cells and integrate their roles in the skeletal cell damage induced by heat stress (HS). Cells were cultured at 37.0 °C or 41.5 °C for 4, 6 or 8 days. The mRNA expression of 24 selenoprotein encoding genes and abundance of 5 selenoproteins were investigated. HS suppressed myogenic differentiation and impaired the development of muscle myotubes. HS down-regulated (*P* < 0.01) mRNA abundance of *MYOD* and *MYOGENIN*, and decreased (*P* < 0.01) MYOGENIN protein expression, HS elevated (*P* < 0.01) HSP70 and (*P* < 0.01) the ratio of BCL-2 to BAX at both mRNA and protein level. Meanwhile, HS up-regulated (*P* < 0.01–0.05) expressions of 18, 11 and 8 selenoprotein encoding genes after 4, 6 and 8 days of hyperthermia, and only down-regulated (*P* < 0.01) *DIO2* after 6 and 8 days of hyperthermia, respectively. Furthermore, HS influenced expression of selenoproteins and up-regulated (*P* < 0.01–0.05) GPX1, GPX4 and SEPN1 after 6 days of HS. The damage to development of mouse skeletal muscle myotubes by HS accompanied with the up-regulation of both selenoprotein encoding genes and proteins, which suggested a potential protective effect of selenoprotein on hyperthermia associated damage in C2C12 cells.

## Introduction

The climate change with increased surface temperature on the earth occurs globally in the past decades. Heat stress (HS) has been is a challenge of the animal industry. HS can be simply defined as a condition in which the animal cannot dissipate excess heat in the body, either produced by itself or absorbed from the environment, to maintain its body thermal balance^1^. The disruption of thermal balance by HS negatively impacts animal’s physiology and performance including decreases in feed intake and milk yield, alterations in milk composition and carcass traits, growth retardation and reproduction disorders^2–6^, which severely influence the animal agriculture. Thus, HS induces financial burden globally^7,8^, and part of the economic distress derives from decreased carcass value. It has been documented over the past 40 years that the pig under HS has reduced muscle mass and increased adipose tissue^9–11^. Rats exposed to heat stress exhibit a subsequent retardation of muscle development^12^.

Skeletal muscle is the major component of edible animal products. Skeletal muscle is differentiated from satellite cells and is highly adaptive to stress due to a remarkable regenerative capability, which is attributed to the high proliferation and differentiation rate of satellite cells. Myogenesis is a two-step process including determination of the muscle lineage committed from satellite cells and differentiation of committed myoblasts to myotubes^13,14^. The C2C12 cells derived from murine skeletal muscle cells is a well-established model to study muscle regeneration and differentiation^15^. In our study, C2C12 cells were used to investigate the response of skeletal muscle cells to heat stress during differentiation.

During differentiation of C2C12 cells, myoblasts undergo remodeling to form mature myotubes in parallel with the increased expression of muscle specific genes^16^. This process requires activation of myogenic regulatory factors (MRF), including myogen termination gene (MYOD), MYOGENIN, MRF4, and myogenic factor 5 (MYF5)^17^. 5′-AMP-activated protein kinase (AMPK) is well known as a sensor for cell energy status^18–20^ and plays an important role in muscle development that the activation of AMPK inhibits myogenesis and hypertrophy of skeletal muscle cells, and decreases muscle mass^21^. Heat stress has been associated with abnormality of cell function, including inhibition of protein synthesis, changes in protein folding and function, alteration in metabolism and membrane fluidity^22,23^. The nucleated cell responds to short period of (non-damaging) stress by synthesizing heat shock proteins (HSPs), a family of stress responsive protein. HSP70 is a well characterized marker of cellular stress responding to heat and other stressors in a variety of organism^24^. Increased cellular content of HSP70 protects cells from stress induced impairment^16^.

Selenium (Se) is a micronutrient essential for animals. Study shows that Se supplementation alleviates the negative effect of HS^25^. Selenium exerts most of its biological functions in the form of selenoproteins, which contain at least one selenocysteine (Sec) in their active center^26^. A total of 25 selenoprotein coding genes have been identified in mammals and 24 in rodents^27,28^. Selenoproteins have been involved in the regulation of redox balance, protection protein from oxidized damage, immunomodulatory, cell apoptosis, protein folding, and degradation of misfolded proteins in endoplasmatic reticulum (ER)^29^. Our previous studies showed that selenoprotein encoding genes were influenced by HS in a porcine small intestinal epithelial cell line (IPEC-J2)^30^, which suggested that they play important roles in cells under HS. However, the metabolic impact of HS on skeletal muscle and expression of selenoprotein encoding genes remain unclear, and it is necessary to explore the impact of HS on expression of selenoproteins using skeletal muscle cells model.

C2C12 isolated from mouse lines by Yaffe and Saxel, which mimics the development of skeletal muscle *in vivo*, representing an excellent model to study myogenic regulation and response to stimuli^14,31^. Therefore, we conducted this to determine (1) impact of HS on myogenic differentiation in C2C12 cells; (2) effect of HS on the gene or protein expression of selenoproteins, myoblast differentiation-related protein, apoptosis-related protein and HSP70; (3) impact of HS on antioxidant attributes of C2C12 cells. The results may help to further explore the potential roles of selenoproteins in skeletal cells faced to HS.

## Results

### Effect of HS on C2C12 cell differentiation

**Figure 1:**
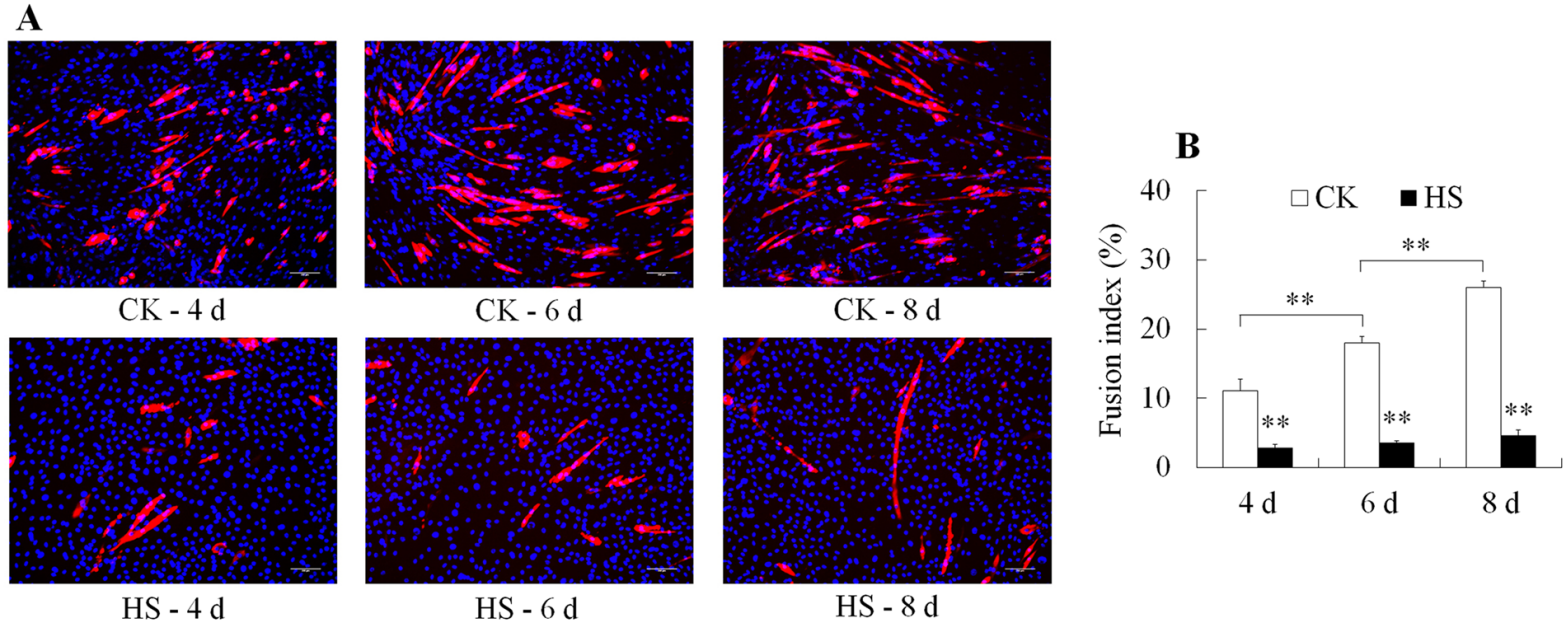
Morphological changes of differentiated C2C12 cells after exposure to heat stress. (**A**) Representative images of differentiated C2C12 subject to HS. Blue color indicated DAPI-stained nuclei and red color indicated stained differentiated myotubes. Bars, 200 µm; (**B**) Changes of fusion index of C2C12 cells after exposure to heat stress. Each column shows means ± SE of 3 independent cultures (*n* = 3). **P < 0.01.

As shown in Fig. S1, serum starvation medium triggered myogenic differentiation as indicated by formation of myotubes after 4 days of induction, while HS impaired development of muscle myotubes that cells became round with dramatically decreased number of myotubes. Compared to control cells, the myotubes incubated at 41.5 °C were poorly formed (Fig. 1A). The fusion index was increased (*P* < 0.01) from 11.1% to 26.0% after 4, 6 and 8 days of incubation in control cells, whereas it was significantly decreased (*P* < 0.01) when cells were incubated at 41.5 °C (Fig. 1B).

### Effect of HS on expression of differentiation-related genes and AMPK genes

**Figure 2:**
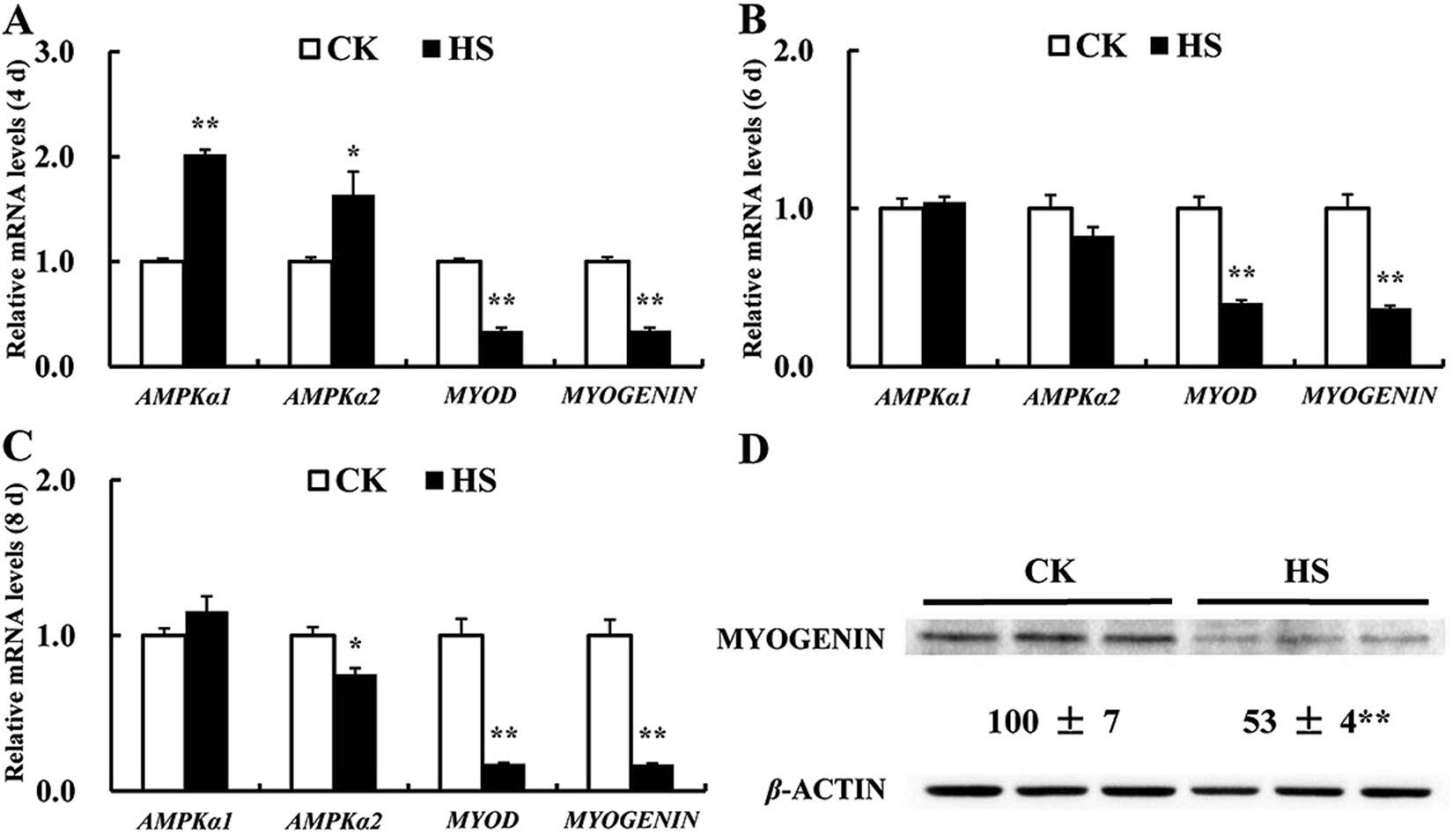
Effect of HS on relative mRNA profiles of *AMPKα1*, *AMPKα2*, *MYOD* and *MYOGENIN* and protein level of MYOGENIN in the differentiated C2C12 cells. mRNA expression at day 4 (**A**); at day 6 (**B**); at day 8 (**C**); Protein level of MYOGENIN at day 6 (**D**). Values are means ± SE (*n* = 6 for genes and 3 for protein). **P < 0.01, *P < 0.05.

We further investigated effect of HS on mRNA and protein expression of *MYOD*, *MYOGENIN*, *AMPKα1* and *AMPKα2* in the differentiating C2C12 cells (Fig. 2). Compared to the CK groups, HS decreased (*P* < 0.01) mRNA expression of *MYOD* by 66%, 60%, 83%, and *MYOGENIN* by 66%, 63%, 83%, in C2C12 cells at day 4, 6, 8, respectively (Fig. 2A–C). HS also decreased (*P* < 0.01) the protein abundance of MYOGENIN by 47% at day 6 (Fig. 2D), confirming HS impairs C2C12 cell differentiation. HS increased *AMPKα1* (*P* < 0.01) and *AMPKα2* (*P* < 0.05) mRNA profiles at day 4 (Fig. 2A), but decreased (*P* < 0.05) mRNA expression of *AMPKα2* at day 8 (Fig. 2C) in differentiated C2C12 cells.

### Effect of HS on expression of HSP70

**Figure 3:**
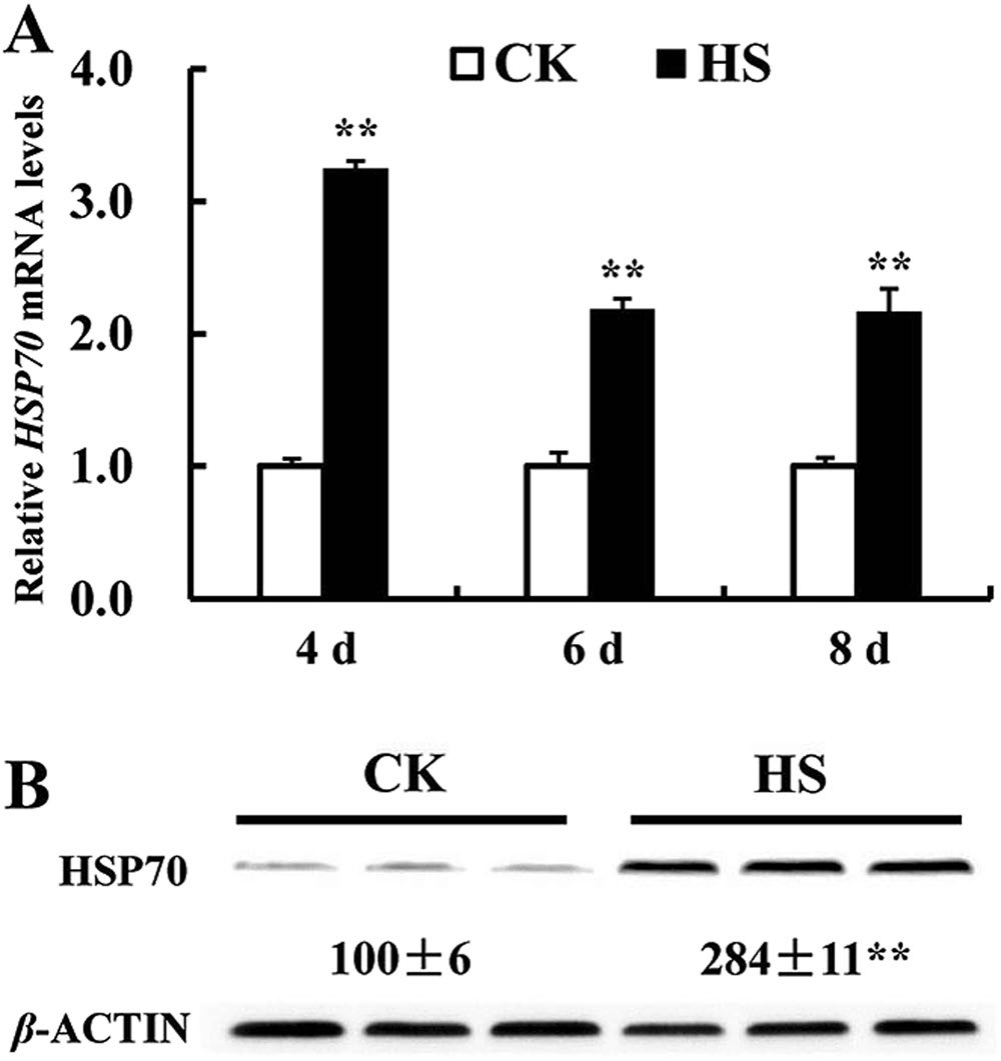
Effect of HS on the mRNA (**A**) and protein (**B**) level of HSP70 in the differentiated C2C12 cells after incubation for 6 day. Values are means ± SE (*n* = 6 for mRNA and 3 for protein). **P < 0.01.

We investigated effect of HS on mRNA and protein levels of HSP70, which is a sensitive cellular indicator for heat stress. As expected, HS increased (*P* < 0.01) both mRNA (Fig. 3A) and protein (Fig. 3B) levels of HSP70 in differentiated C2C12 cells, respectively.

### Effect of HS on expression of selenoproteins

**Figure 4:**
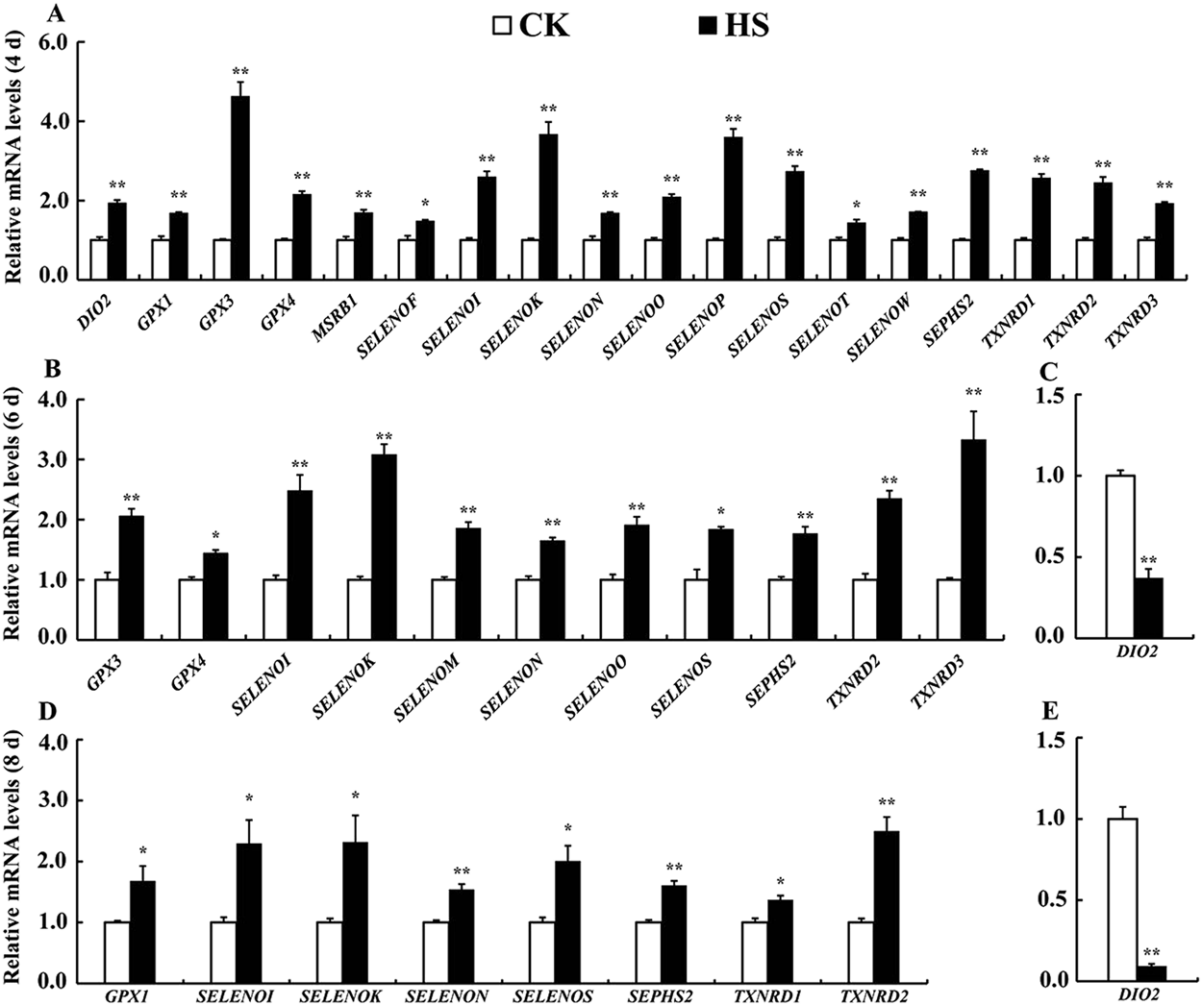
Effect of HS on relative mRNA levels of selenoprotein encoding genes in the differentiated C2C12 cells. (**A**) The up-regulated selenoprotein genes under HS for 4 days; (**B**) The up-regulated selenoprotein genes under HS for 6 days; (**C**) The down-regulated selenoprotein genes under HS for 6 days; (**D**) The up-regulated selenoprotein genes under HS for 8 days; (**E**) The down-regulated selenoprotein genes under HS for 8 days. Data are means ± SE (*n* = 6). **P < 0.01, *P < 0.05.

We explored effect of HS on mRNA abundance of 24 selenoprotein encoding genes in the myogenic differentiated C2C12 cells. HS increased (*P* < 0.05) mRNA profiles of 18 selenoprotein encoding genes (*DIO2*, *GPX1*, *GPX3*, *GPX4*, *MSRB1*, *SELENOF*, *SELENOI*, *SELENOK*, *SELENON*, *SELENOO*, *SELENOP*, *SELENOS*, *SELENOT*, *SELENOW*, *SEPHS2*, *TXNRD1*, *TXNRD2*, *TXNRD3*) at the early stage (day 4) (Fig. 4A). With prolonged HS challenge, the number of up-regulated selenoprotein encoding genes decreased. HS led to increases in mRNA expression of 11 selenoprotein encoding genes (*GPX3*, *GPX4*, *SELENOI*, *SELENOK*, *SELENOM*, *SELENON*, *SELENOO*, *SELENOS*, *SEPHS2*, *TXNRD2*, *TXNRD3*) at day 6 (Fig. 4B) and 8 genes (*GPX1*, *SELENOI*, *SELENOK*, *SELENON*, *SELENOS*, *SEPHS2*, *TXNRD1*, *TXNRD2*) at day 8 (Fig. 4D), respectively. Interestedly, *DIO2* was up-regulated (*P* < 0.01) at early stage (day 4) of HS (Fig. 4A), while it was down-regulated (*P* < 0.01) at late stage (day 6 and 8) of HS (Fig. 4C,E). Furthermore, *DIO2* was the only selenoprotein gene that was down-regulated in differentiated C2C12 cells under HS. The profiles of selenoprotein encoding genes are shown in the Table S2.

**Figure 5:**
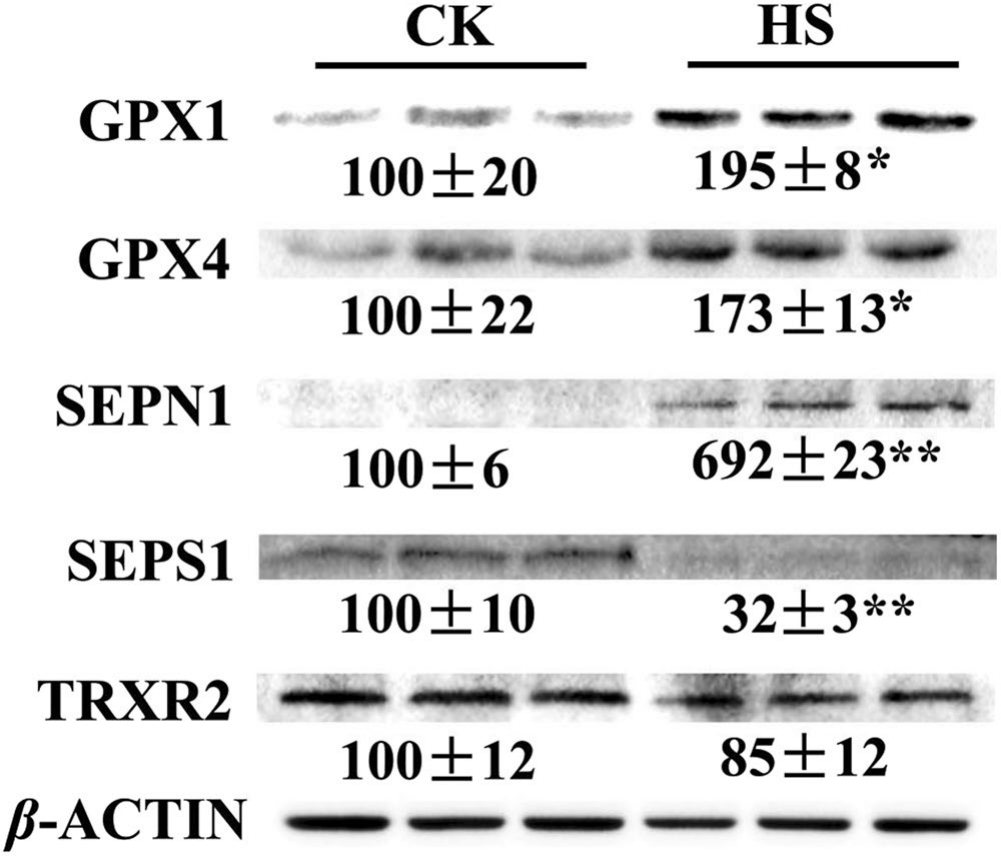
Effect of HS on the protein levels of GPX1, GPX4, SEPS1, SEPN1 and TRXR2 in the differentiated C2C12 cells after incubation for 6 day. Values are means ± SE (*n* = 3). **P < 0.01, *P < 0.05.

We also investigated effect of HS on protein expression of 5 selenoproteins (GPX1, GPX4, SEPS1, SEPN1, and TRXR2) at day 6. Among those selenoproteins investigated, GPX1 and GPX4 have a higher distribution in skeletal tissues and SEPN1 is a selenoprotein relating to muscle development. As shown in Fig. 5, HS increased GPX1 (*P* < 0.05), GPX4 (*P* < 0.05) and SEPN1 (*P* < 0.01) protein abundance, and decreased (*P* < 0.01) SEPS1 abundance while exhibited no effect on TRXR2 (*P* > 0.05) in the differentiated C2C12 cells. The limited availability of antibodies in our Lab prevents us from exploring more selenoproteins in the present study.

### Effect of HS on cell apoptosis

**Figure 6:**
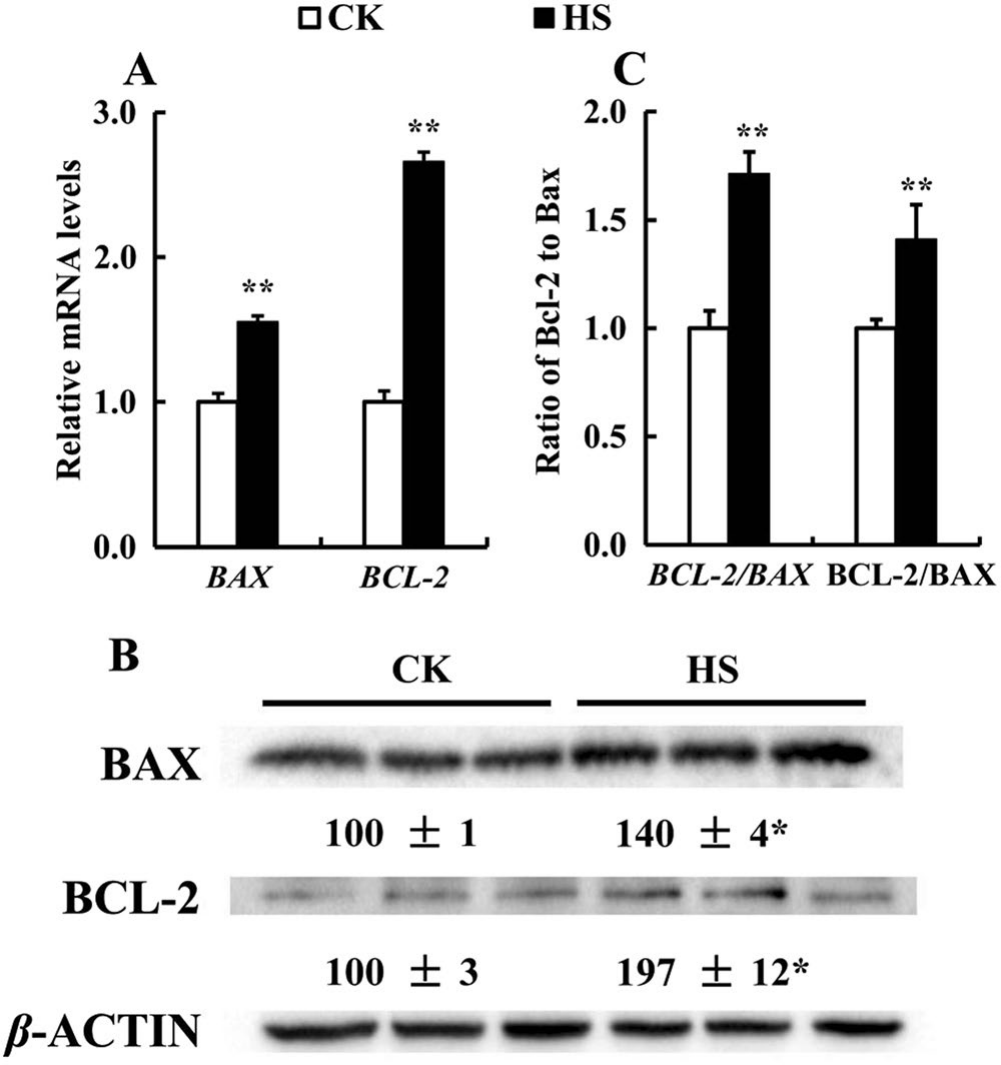
Effect of HS on the mRNA and protein levels of BAX and BCL-2 in the differentiated C2C12 cells after incubation for 6 day. (**A**) The mRNA abundance of the *BAX* and *BCL-2* (*n* = 6). (**B**) The protein levels of BAX and BCL-2 (*n* = 3). (**C**) The mRNA and protein ratio of BCL-2 to BAX. Values are means ± SE. **P < 0.01, *P < 0.05.

To determine whether HS induces apoptosis in differentiated C2C12 cells, we investigated effect of HS on expression of BCL-2 and BAX (Fig. 6). The results showed that HS increased both mRNA abundance (*P* < 0.01) (Fig. 6A) and protein levels (*P* < 0.05) (Fig. 6B) of BCL-2 and BAX. The ratio of BCL-2/BAX at mRNA and protein level (Fig. 6C) was also significantly increased (*P* < 0.01) by HS.

### Effect of HS on antioxidant attributes in C2C12 cells

To determine whether HS induces oxidative stress in differentiated C2C12 cells, effect of HS on activity of glutathione peroxidase (GSH-Px), total superoxidase dismutase (T-SOD), and concentration of malondialdehyde (MDA) in differentiated C2C12 cells were investigated, and results are shown in Table 1. Compared to control cells, HS challenge for 6 days significantly increased (*P* < 0.01) the activity of T-SOD while had no effect on activity of GSH-Px. MDA is an indicative for oxidative stress in cells or organism. The results showed that HS decreased (*P* < 0.01) the levels of MDA in C2C12 cells.

**Table 1:** Effect of HS on antioxidant measurements.

Measures	CK	HS	*P* value
GSH-Px (U/mg prot)	5.54 ± 1.09	6.86 ± 0.42	0.14
MDA (nmol/mg prot)	1.90 ± 0.09	1.54 ± 0.07	<0.01
T-SOD (U/mg prot)	3.35 ± 0.14	30.88 ± 1.57	<0.01

Values are means ± SE (*n* = 6).

## Discussion

In this study, our target was to investigate effect of HS on the expression of selenoproteins in differentiating C2C12 mouse myoblast. Firstly, we investigated the effect of HS on myogenic differentiation of C2C12 cells. HS impaired the differentiation of C2C12 cells as shown by the suppression of myotube formation in a hyperthermia condition (Fig. 1). Similar results were reported in mouse study that HS impeded the development of myotube in skeletal muscles^16^. Myotubes were poorly formed when primary human skeletal muscle culture cells, human skeletal muscle myoblasts (HSMMs), and C2C12 mouse myoblasts were cultured at 41 °C^32^.

We investigated expression of two myoblast differentiation-related genes. MYOD is essential for skeletal muscle differentiation^33^ through mediating the expression of some muscle-specific genes^34^. Previous study showed the absence of MYOGENIN resulted in a deficiency of muscle fiber despite muscle cell migration and commitment^31^. In our study, HS decreased expressions in both mRNA and protein levels of MYOD and MYOGENIN, indicating that HS suppressed the myogenic differentiation of C2C12 cells. We also investigated expression of two *AMPK* genes. Interestingly, we found HS increased the mRNA expression of *AMPKα1* and *AMPKα2* at day 4, while decreased *AMPKα2* at day 8. The up-regulation of *AMPKα1* and *AMPKα2* at early stage of HS (at day 4) may reflect an increased energy requirement for adaption of metabolism and cell survival. With prolonged HS, cells gradually lost the adaptive function as cell impairment occurred (Fig. 2C).

Heat shock proteins are considered as a cellular thermometer, which is frequently used to evaluate HS response^35^. HSPs are expressed globally in a variety of species and are required for cell survival under stress^36^. The previous studies showed a significant increase in the induction of HSPs, mainly HSP70 and HSP90, in different tissues and cells under HS^37,38^. Increased cellular HSPs can provide cytoprotection against subsequent stresses^16^. HSP70 is the most ubiquitous chaperones and is highly conserved in all organisms^39^. Thus, it has been frequently used to characterize stress response to heat and other stressors in different organisms^24,40^. It was not surprising that HS increased gene and protein expression of HSP70 (*P* < 0.01) in the differentiated C2C12 cells (Fig. 3), which was consistent with previous studies^30,41^.

Selenoprotein encoding genes encode for selenocysteine-containing proteins (selenoproteins), which are involved in a variety of functions including redox homeostasis regulation^28^. However most of their functions are still unknown. Our previous study showed that both mRNA and protein expression of selenoprotein encoding genes were influenced by HS for 24 h in IPEC-J2 cells, and 4 selenoprotein genes (*GPX3*, *DIO2*, *SELENOK*, *SELENOS*) were up-regulated (*P* < 0.05) and six selenoprotein genes (*GPX2*, *GPX6*, *TXNRD1*, *SELENOH*, *SELENOM*, *MSRB1*) were down-regulated (*P* < 0.05 or as indicated) in IPEC-J2 cells by HS^30^. Interestingly, in this study, selenoprotein encoding genes (except *DIO2*) were globally up-regulated by HS in C2C12 cells, which suggesting their potential roles against HS-induced cell damage (Fig. 4). The numbers of these up-regulated genes decreased from 18 to 8 genes from day 4 to day 8 indicating decreased metabolism with exposure duration of HS. It was reported that genes related to cell survival will be turned on, while more unessential genes may be turned off under stress conditions^42^.

Among those selenoproteins influenced by HS, GPXs contribute to antioxidant system in mammals^43^. GPX1 deficiency is correlated with increased susceptibility to oxidative stress^44^. The increased expression of *GPX3* may contribute to detoxify reactive oxygen species (ROS) such as phospholipid hydroperoxide and hydrogen peroxide induced by HS^45^. In this study, HS increased expression of *GPX1*, *GPX3*, and *GPX4* in the differentiated C2C12 cells, indicating the potential protective effects of these selenoproteins in muscle cells against HS.

SELENOK, SELENOM and SEPS1 are endoplasmic reticulum (ER) transmembrane proteins. SELENOK is an ER stress-regulating protein, which modulates cellular redox balance^46,47^. SELENOM acts as a thiol-disulfide oxidoreductase involved in protein folding^48^. SEPS1 induces production of inflammatory cytokines and protects the cell compartment from oxidative stress^49^. The up-regulation of *SELENOS*, *SEPHS2* and *SELENOK* in our study suggested an important role of these selenoproteins in protecting cells from the damage of HS. Although mRNA expression of *SELENOS* was up-regulated, protein level of SEPS1 was down-regulated when cells were challenged with HS for 6 days (Fig. 5). SEPN1 has been involved in muscle physiology as a key regulator of satellite function^50–52^. SEPN1 shows a high expression during the proliferation of fibroblast and myoblast, but it decreases when myoblasts differentiate into myotubes^53^. Absence of SEPN1 was associated with high susceptibility to H_2_O_2_-induced oxidative stress, leading to cell death^54^. The increased SEPN1 expression by HS in differentiated C2C12 cells suggested SEPN1 may protect C2C12 cells from HS.

Thioredoxin (TRX) is an antioxidant that reduces oxidized moieties^55^. Thioredoxin reductases (TRXRs) are crucial to regenerate reduced TRX to maintain balance between reduced and oxidized molecules^56,57^. The up-regulation of *TXNRD1*, *TXNRD2* and *TXNRD3* in C2C12 cells suggested that TRX might contribute to maintain the redox balance in muscle cells under HS, these may partly explained why MDA were not increased in the stressed cells (Table 1). The protein levels of TRXR2 were not decreased by HS at the 6^th^ day (Fig. 5), implying a physiological necessity for a constant expression of TRXR2 to deal with HS.

Iodothyronine deiodinase 2 (DIO2) converts thyroxine (T4) to bioactive 3,5,3′-tri-iodothyronine (T3) to initiate the action of thyroid hormone^58^. DIO family is comprised of 3 isoforms, DIO1, DIO2 and DIO3. *DIO2* was the only selenoprotein encoding gene that was down-regulated by HS in C2C12 cells. It has reported that T3 generated from T4 by DIO2 is key to maintain C2C12 cells differentiation, and T3 were essential for the enhanced transcription of MyoD^59^. In the present study, decreases expression of *DIO2*, MYOD and MYOGENIN is consistent with the low level of differentiation under HS. Thyroid hormone improves critical protein synthesis^60^, however cells may have to decrease cell metabolism to survive with extended HS^61^, which may partly explain the down-regulation of *DIO2* (*P* < 0.01) at late stage (Fig. 4C,E).

Hyperthermia investigations at cellular level showed some types of cell underwent apoptosis in response to heat stress^22^. BCL-2 genes play important roles in regulating apoptosis, including antiapoptotic protein BCL-2 and proapoptotic protein BAX^62^. The ratio of BCL-2 to BAX represents the level of apoptosis^63^. We found that HS increased (*P* < 0.01) ratios of *BCL-2*/*BAX* at both mRNA and protein level in C2C12 cells, which were consistent with previous study in C2C12 cells^64^. Increased BAX may indicate apoptosis, and increase in the BCL-2/BAX ratio would indicate the anti-apoptosis. The up-regulation of selenoproteins may contribute to anti-apoptosis and prevent cells underwent apoptosis by HS. ROS generated through a variety of extracellular and intracellular actions has drawn attention as novel signal mediator involved in growth, differentiation, progression, and death of cells^65^. A previous study has shown that HS caused overproduction and accumulation of ROS, leading to the impairment of cells^66^. Chicken exposed to HS resulted in a significant increase in activities of SOD, CAT and GPx^67^. In the present study, HS greatly increased activity of T-SOD in C2C12 cells, while decreased levels of MDA (Table 1). GSH-Px showed no response, however it increased in value (*P* = 0.14) by HS. MDA is used as a biomarker to measure the level of oxidative stress in an organism^68^, and the decreased levels of MDA indicate cells were not in an oxidative stress condition. Our previous results shows that HS has limited effect on antioxidant measurements in porcine IPEC-J2 cells, and MDA exhibits a decreasing tendency in HS stressed cells^30^. It seems oxidative stress is not the major factor for C2C12 cells damages induced by HS, possibly the up-regulation of selenoprotein encoding genes contribute to preventing the increasing of MDA.

In summary, HS impairs the differentiation of C2C12 cells and induces selenoprotein responses. Although information available concerning the relations between selenoproteins response and HS in skeletal muscle was still limited, studies yet elucidated was that the increased mRNA and protein expression of HSP70 protected cells from heat stress. Therefore, many selenoprotein encoding genes or proteins were up-regulated in C2C12 cells under HS, which implied the potential protective effect of these selenoproteins against the impairment induced by hyperthermia. The results may also implied the potential of these selenoproteins act as target genes or protein be used to further investigate the effect of husbandry temperature on meat quality or production.

## Materials and Methods

### Cell culture

The C2C12 mouse myoblast cell line was maintained in medium (DMEM; Gibco, USA) containing 1% penicillin-streptomycin (Gibco, USA) and 10% (v/v) fetal bovine serum (FBS; Gibco, USA). 1 × 10^5^ cells/well of cells were seeded in 12-well plates and cultured at 37 °C under 5% CO_2_. After reaching to 80% confluence, cells were divided into two groups: cells in control group (CK) were cultured at 37 °C, while cells in HS group (HS) were exposed to a hyperthermia condition at 41.5 °C. Meanwhile, differentiation were triggered by replacing 10% FBS to 2% horse serum (Gibco, USA), and cells were cultured for another 4, 6 or 8 days. The differentiation media were changed every two days.

### Immunofluorescence staining

After HS treatment for 4, 6, 8 days during differentiation, cells were washed with warm PBS (37 °C) and fixed in 4% paraformaldehyde at room temperature for 30 min and then applied for immunofluorescent staining for myotubes and 4,6-diamidino-2-phenylindole (DAPI) staining for nuclei as described by Yamaguchi *et al*.^32^. The primary antibody was mouse anti-MyHC (1:200; Zen BioScience, China) and the secondary antibody was fluorescence-conjugated goat anti-mouse IgG (1:1000; Millipore, USA). The immunofluorescence stained cells were examined with fluorescent microscope (DMI 4000B; Leica, Germany). The fusion index was defined and determined according to Yamaguchi *et al*.^32^.

### Real-time quantitative PCR analyses

After HS treatment for 4, 6, 8 days during differentiation, the cells were harvested for total RNA extraction using TRIzol (Invitrogen, USA). Two wells of cells were pooled together and in each treatment six samples were collected (*n* = 6). The qPCRs procedure and relative mRNA abundance quantification were conducted as previously described using 2^−ΔΔCt^ method^30,69^. For each measurement, all samples were run on the same plate. Primer Express 3.0 (Applied Biosystems, USA) was used for primers design and primers for 4 myogenic differentiation-related genes (*AMPKα1*, *AMPKα2*, *MYOD* and *MYOGENIN*), 24 selenoprotein encoding genes, *HSP70*, 2 apoptosis-related genes (*BAX* and *BCL-2*), and 2 reference gene (*β-ACTIN* and *GAPDH*) are presented in Table S1.

### Western blot analyses

Cell culture and HS treatment were conducted as mentioned above, and cells for protein extraction were grown in 6-well plates. After HS treatment for 6 days, cells were harvested and protein was extracted using RIPA lysis buffer^30^. Each treatment contain three replicates (*n* = 3) and four wells of cells were pooled together for each replicate. Western blot was processed as described previously by our group^30^. The primary antibodies included MYOGENIN (1:800; Zen BioScience, China), HSP70 (1:5000; Abcam, USA), GPX1 (1:1000; Zen BioScience, China), GPX4 (1:2000; Zen BioScience, China), SEPS1 (1:800; Zen BioScience, China), SEPN1 (1:800; Proteintech, China), TRXR2 (1:800; Zen BioScience, China), BAX (1:5000; Proteintech, China), BCL-2 (1:800; Proteintech, China), and *β*-ACTIN (1:5000; Millipore, USA). The secondary antibodies were horseradish peroxidase-linked goat anti-rabbit IgG (1:10000; CST, USA) or goat anti-mouse IgG (1:20000; Millipore, USA). Electrochemiluminescence (ECL) was used to detect a specific protein signal and western blot bands were analyzed using Image Lab™ software system (Bio-Rad, USA).

### Enzyme activity assays

After HS treatment for 6 days, cells were harvested and digested with 0.25% trypsin. Samples (*n* = 6) were prepared as described previously^30^. Activity of GSH-Px, T-SOD and concentration of MDA were determined using corresponding kit according to the manufacturer’s instructions. Kits for GSH-Px (No. A005), T-SOD (No. A001–1–1) and MDA (No. A003-4) were purchased from Jiancheng Bioengineering, China, respectively. Protein concentration was determined by the BCA method. The optical density (OD) values were measured with an UV-visible spectrophotometer (SpectraMax 190, MD, USA).

### Statistical analysis

Independent *t*-test (SPSS for Windows 13.0, Chicago, IL) was used to determine the influence of HS on investigated index in C2C12 cells. Data are presented as means ± SE and significance level is set at *P* < 0.05.

## Electronic supplementary material

Supporting materials
