# Supplementary material for: Damage to the myogenic differentiation of C2C12 cells by heat stress is associated with up-regulation of several selenoproteins
Source: Sci Rep. 2018 Jul 13;8:10601. doi: 10.1038/s41598-018-29012-6 (PMC6045685; doi:10.1038/s41598-018-29012-6)
Supplement: Supplementary file 1 — Supporting materials [file 41598_2018_29012_MOESM1_ESM.pdf]

Damage to the myogenic differentiation of C2C12 cells by heat stress is associated with up-regulation of several selenoproteins

Jiayong Tang[1,2$], Aihua He[1$], Hui Yan[3],Gang Jia[1], Guangmang Liu[1], Xiaoling Chen[1], Jingyi Cai[1], Gang Tian[1], Haiying Shang[1], and Hua Zhao[1,2]*

[1]Animal Nutrition Institute, Sichuan Agricultural University, Chengdu, Sichuan 611130, China;

[2]Trace Element Research Center, Sichuan Agricultural University, Chengdu, Sichuan 611130, China;

[3]Department of Animal Sciences, Purdue University, West Lafayette, IN 47907, U.S.A.

*****Correspondence: zhua666@126.com; Tel.: +86 28 8629-0922; Fax: +86 28 8629-0922

Supplementary Figure legends

Fig.S1. Microscope inspection of the myogenic differentiated C2C12 cells subject to heat stress.

Table S1 Primers used for the Q-PCR of the target and reference genes

Protein	Gene	Accession number	Primer sequence (from 5' to 3')
		**Selenoprotein genes**	
DIO1	*DIO1*	NM_007860.3	F: AAGCAAGAGGCAGGCATGTT<br>R: CGGCCAGAAAAGTGTTTCCA
DIO2	*DIO2*	NM_010050.2	F:GGAATGTTGGCCAGTTTTGTTT<br>R:TGGTTACATGGGCTGGTGAGT
DIO3	*DIO3*	NM_172119.2	F: AGGTGTCTGAGTTGCGCACTT<br>R: TGGCCTAGTACCATGCAACTGT
GPX1	*GPX1*	NM_008160.6	F: AGGCTCACCCGCTCTTTACC<br>R: GGGTCGTCACTGGGTGTTG
GPX2	*GPX2*	NM_030677.2	F: TGTGACGTCAATGGGCAGAA<br>R: AGGGCAGCTTGTCTTTCAGGTA
GPX3	*GPX3*	NM_008161.3	F: ACAGGAGCCAGGCGAGAA<br>R: CCACCTGGTCGAACATACTTGA
GPX4	*GPX4*	NM_001037741.3	F: GCCGGCTACAACGTCAAGTT<br>R: GGCATCGTCCCCATTTACAC
MSRB1	*MSRB1*	AF195142.1	F: CAGCCTCAGTCACCGAATGA

			R: ACCACCCTGGCTGGCATA
SEP15	*SELENOF*	NM_053102.2	F: TGGACGACAACGGGAACAT<br>R: CCACACTGTCTGTGTTCCACTTG
SELH	*SELENOH*	NM_001033166.2	F: ATTCCCGGCTGCTGGTTT<br>R: GGCGCGTTGGTGGAATAA
SELI	*SELENOI*	NM_027652.2	F: GCTTTGGGAGCAGTGTGCTAT<br>R: AGCTCTGCTCCAGCAAGATCA
SELK	*SELENOK*	NM_019979.2	F: GGGTAGGATCAGTCACCTTCGT<br>R: TTCCTCATCCACCAGCCATT
SELM	*SELENOM*	NM_053267.2	F: GGAGACCTGTGGAGGATGACA<br>R: TCGGTGACAAAGGCCTTCAC
SEPN1	*SELENON*	NM_029100.2	F: ACCGGATGGCCACCAGTT<br>R: GGTCAGCCGTTCAAGCTGTT
SELO	*SELENOO*	NM_027905.2	F: CCCCAGGTATGCAAGTGGAA<br>R: AGTGGCAGTTCAGGCTCCAA
SELP	*SELENOP*	X99807.1	F: CAGGGTCTGCAATTGCTTGA<br>R: GAAAAGCCCCTGTCAGCTACA
SEPS1	*SELENOS*	NM_024439.3	F: TGTTAAGCGGCAAGAGGCTTT<br>R: GGGCATTTAGATCTTCCTGCAT
SELT	*SELENOT*	NM_001040396.2	F: TGCACTCGCATTCGTGACA<br>R: ACTGGAGCTCACCGCATTG
SELV	*SELENOV*	NM_175033.3	F: GCTGCTAGCGCTCTCTTGAAG<br>R: GTGGATCGAGGGTTTCTGATTT
SEPW1	*SELENOW*	AF015284	F:GCCGTTCGAGTCGTGTATTGT<br>R:TCTCCTTGAGCTGGAGGTACTTG
SPS2	*SEPHS2*	NM_009266.3	F: CGTTGGCATCGTGGAGAAG<br>R: CGCGAGGCTTGTCAATGAT
TRXR1	*TXNRD1*	BC037643.1	F: CACAAACAGCGAGGAGACCAT<br>R: TTCCTACCGCCAGCAACACT

TRXR2	*TXNRD2*	NM_013711.3	F: GTTCACGGTGGCGGATAGG<br>R: GCTCCCTCATGCATACCATCTT
TRXR3	*TXNRD3*	C076605.1	F: GGGACATACTGGACGGCAAA<br>R: TAGCAGCTTGCCTGCCTGTA
**Myogenic differentiation-related genes**			
AMPKα1	*AMPKα1*	AY885266.1	F: GGCACACCCTGGATGAATTAA<br>R: TGCCTTCCGTACACCTTGGT
AMPKα2	*AMPKα2*	BC138566.1	F: TCGCAGACAGCCCCAAAG<br>R: TTGGGCTTCGTTGTGTTGAG
HSP70	*HSP70*	EU622851.1	F: CCACCCGCATCCCAAAG<br>R: TGTTGAGATCCCGTCCATTAAA
MYOD	*MYOD*	M84918.1	F: CAGCATCACGGTGGAGGATA<br>R: CAGTTGGGCATGGTTTCGT
MYOGENIN	*MYOGENIN*	BC048683	F: GGCTGCCTAAAGTGGAGATCCT<br>R: AGGCCTGTAGGCGCTCAAT
**Apoptosis-related genes**			
BAX	*BAX*	L22472.1	F: AGGATGCGTCCACCAAGAAG<br>R: GCTATCCAGTTCATCTCCAATTCG
BCL-2	*BCL-2*	BC095964.1	F: TGGGATGCCTTTGTGGAACT<br>R: CAGCCAGGAGAAATCAAACAGA
**Housekeeping control genes**			
β-ACTIN	*β-ACTIN*	NM_007393.5	F: ACCAGTTCGCCATGGATGAC<br>R: TGCCGGAGCCGTTGTC
GAPDH	*GAPDH*	GU214026.1	F: GGGAAGCCCATCACCATCT<br>R: CGGCCTCACCCCATTTG

Table S2 Effects of HS on relative mRNA levels of selenoprotein genes in C2C12 cells

Differentiation time	Gene	CK[1]	HS[1]	*P* value
4 day	*DIO1*[2]	-	-	-
	DIO2	1.00±0.08	1.93±0.08	< 0.001
	DIO3	-	-	-
	GPX1	1.00±0.10	1.68±0.03	< 0.001
	GPX2	-	-	-
	GPX3	1.00±0.03	4.62±0.37	< 0.001
	GPX4	1.00±0.04	2.15±0.08	< 0.001
	MSRB1	1.00±0.09	1.69±0.08	< 0.001
	SELENOF	1.00±0.11	1.48±0.03	0.013
	SELENOH	1.00±0.10	0.76±0.03	0.110
	SELENOI	1.00±0.06	2.59±0.15	< 0.001
	SELENOK	1.00±0.04	3.66±0.32	< 0.001
	SELENOM	1.00±0.06	1.12±0.02	0.163
	SELENON	1.00±0.10	1.677±0.03	0.001
	SELENOO	1.00±0.06	2.08±0.08	< 0.001
	SELENOP	1.00±0.05	3.59±0.21	< 0.001
	SELENOS	1.00±0.07	2.73±0.14	< 0.001
	SELENOT	1.00±0.07	1.43±0.09	0.014
	SELENOV	-	-	-
	SELENOW	1.00±0.06	1.71±0.01	< 0.001
	SEPHS2	1.00±0.04	2.75±0.03	< 0.001
	TXNRD1	1.00±0.06	2.57±0.11	< 0.001
	TXNRD2	1.00±0.06	2.44±0.15	< 0.001
	TXNRD3	1.00±0.07	1.92±0.04	< 0.001
6 day	*DIO1*	-	-	-
	DIO2	1.00±0.03	0.37±0.06	< 0.001
	DIO3	-	-	-

	GPX1	1.00±0.09	1.31±0.15	0.174
	GPX2	-	-	-
	GPX3	1.00±0.12	2.06±0.12	0.001
	GPX4	1.00±0.05	1.44±0.06	0.010
	MSRB1	1.00±0.12	1.17±0.07	0.437
	SELENOF	1.00±0.07	0.87±0.04	0.324
	SELENOH	1.00±0.11	0.94±0.17	0.626
	SELENOI	1.00±0.07	2.48±0.26	0.002
	SELENOK	1.00±0.05	3.08±0.18	< 0.001
	SELENOM	1.00±0.05	1.85±0.10	0.002
	SELENON	1.00±0.06	1.65±0.06	0.003
	SELENOO	1.00±0.09	1.91±0.14	0.005
	SELENOP	1.00±0.10	1.44±0.07	0.101
	SELENOS	1.00±0.17	1.84±0.04	0.011
	SELENOT	1.00±0.13	0.98±0.07	0.900
	SELENOV	-	-	-
	SELENOW	1.00±0.09	1.20±0.04	0.236
	SEPHS2	1.00±0.05	1.76±0.12	0.005
	TXNRD1	1.00±0.04	0.78±0.09	0.104
	TXNRD2	1.00±0.10	2.35±0.14	< 0.001
	TXNRD3	1.00±0.03	3.33±0.47	0.002
**8 day**	*DIO1*	-	-	-
	DIO2	1.00±0.07	0.09±0.02	< 0.001
	DIO3	-	-	-
	GPX1	1.00±0.03	1.68±0.24	0.053
	GPX2	-	-	-
	GPX3	1.00±0.06	0.93±0.03	0.414
	GPX4	1.00±0.05	1.35±0.14	0.101
	MSRB1	1.00±0.05	1.55±0.25	0.293

SELENOF	1.00±0.06	1.02±0.16	0.915
SELENOH	1.00±0.04	1.33±0.15	0.127
SELENOI	1.00±0.09	2.30±0.38	0.029
SELENOK	1.00±0.07	2.32±0.44	0.042
SELENOM	1.00±0.06	0.93±0.13	0.593
SELENON	1.00±0.03	1.54±0.09	0.002
SELENOO	1.00±0.05	1.33±0.13	0.088
SELENOP	1.00±0.04	0.92±0.12	0.649
SELENOS	1.00±0.08	2.01±0.25	0.016
SELENOT	-	-	-
SELENOV	-	-	-
SELENOW	1.00±0.05	1.06±0.10	0.708
SEPHS2	1.00±0.04	1.61±0.07	0.001
TXNRD1	1.00±0.07	1.37±0.07	0.014
TXNRD2	1.00±0.06	2.50±0.23	0.002
TXNRD3	1.00±0.10	1.18±0.09	0.177

[1]Values are mean ± SE (n = 6).

[2]These genes without values indicate they provided results that were too close to background to be interpreted or reported.

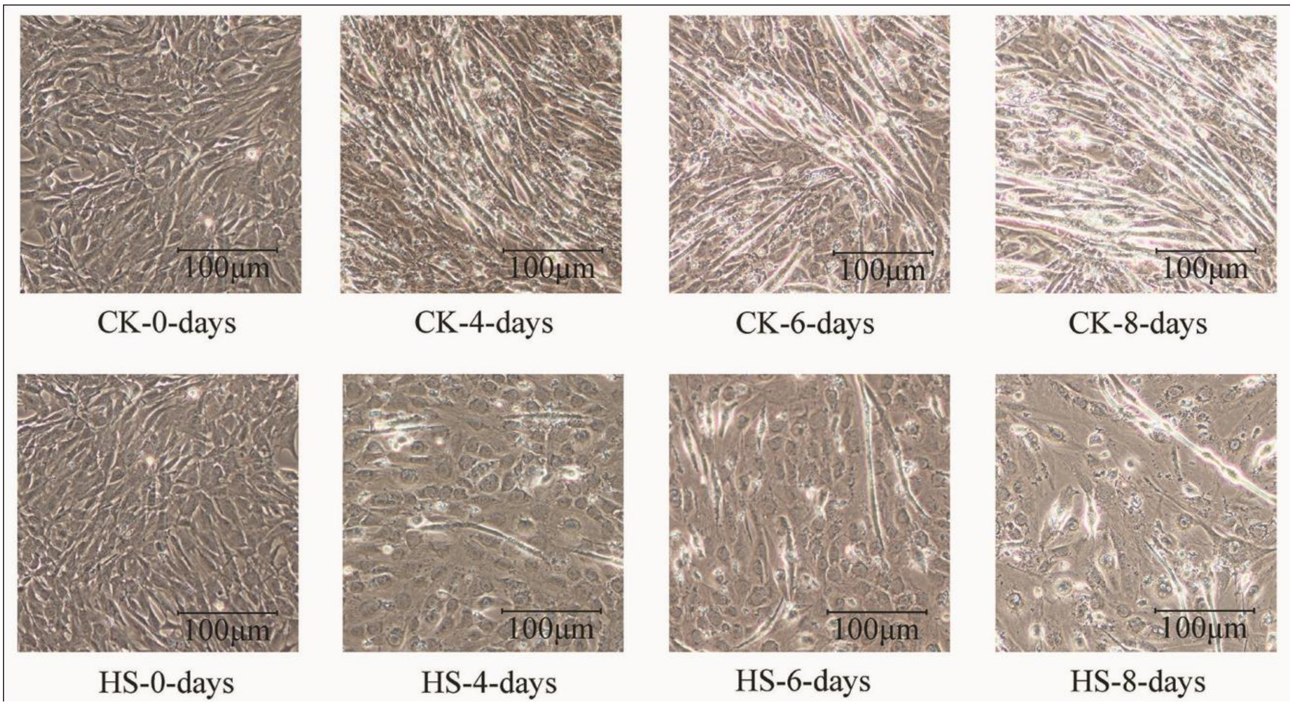

Fig.S1. Microscope inspection of the myogenic differentiated C2C12 cells subject to heat stress.
